# Assessing the Ability of Non-dermatology Physicians to Recognize Urgent Skin Diseases

**DOI:** 10.7759/cureus.37823

**Published:** 2023-04-19

**Authors:** Abdulaziz G Aljohani, Mohammed H Abduljabbar, Jehad Hariri, Bader S Zimmo, Maan A Magboul, Saud M Aleissa, Ahmed Baabdullah, Abdulsalam Alqutub, Khalid Alafif, Hassan Faidah

**Affiliations:** 1 Medicine, King Abdulaziz University Faculty of Medicine, Jeddah, SAU; 2 Dermatology, King Abdulaziz University Hospital, Jeddah, SAU

**Keywords:** improve patient care, appropriate management, urgent referral, initial judgment, misdiagnose, physicians, non-dermatologists, urgent skin conditions, dermatological emergencies, cross-sectional study

## Abstract

Introduction

Many patients present to the emergency department with skin conditions that are not true dermatologic emergencies. Urgent skin conditions are uncommon. Because these conditions are rare, they can be sometimes challenging to diagnose. Few works of literature discussed the accuracy of non-dermatologists' initial judgment on dermatologic conditions concluding that non-dermatologists misdiagnose many common and uncommon skin conditions. Because the study was never done in our region, we aim to conduct an online assessment using a questionnaire assessing the ability of non-dermatologists to recognize urgent skin diseases at King Abdulaziz University Hospital (KAUH) in Jeddah, Saudi Arabia.

Methods

A cross-sectional study was conducted. Non-dermatologist physicians were contacted through their verified emails, provided by the secretaries of each department and the academic affairs unit. The questionnaire consisted of two main sections, the first section covered demographics, specialty, and academic level. The second part had eight questions, each containing a brief case scenario about an urgent dermatological condition with a picture of the condition attached. Participants were required to answer the questions and assess on a scale from one to 10 how confident they were about their answers. The responses were collected and analyzed.

Results

Out of all 161 responses, this study included 93 male physicians (57.8%) and 68 female physicians (42.2%). The mean age in the study was approximately 45 ± 3 years. This study showed that the percentage of accuracy by non-dermatologists in diagnosing urgent skin diseases given the typical presentation of the condition was (61.33%); nevertheless, the percentage decreased when it was recalculated in relation to the full level of confidence to (25.3%). Herpes zoster appeared to be the most recognizable urgent skin disease, and Pemphigus vulgaris was the least recognizable one.

Conclusion

This study shows that it is difficult for physicians to recognize some urgent skin diseases, which affects offering the optimum health care for the patients. Moreover, more dermatology-focused courses are needed to strengthen the knowledge about dermatological diseases.

## Introduction

Many patients present to the emergency department with skin conditions that are not true dermatologic emergencies [1]. Urgent skin conditions caused by allergic drug reactions, severe immune-mediated diseases or infections, such as Steven Johnson syndrome, toxic epidermal necrolysis, Pemphigus vulgaris, or Staphylococcal scalded skin syndrome, are uncommon. Because these conditions are rare, they can be sometimes challenging to diagnose. Nevertheless, when these urgent conditions present at a hospital, both dermatologists and non-dermatologists are responsible for their care. In Saudi Arabia, dermatologists are available in big cities, but many smaller cities and rural areas lack specialized dermatologists [2]. Therefore, in those cities, non-dermatology physicians must recognize and manage many skin diseases and assess whether these conditions need immediate or urgent referral to dermatologists.

Few works of literature covered this topic throughout different decades. In different countries, some of these studies focused only on diagnosing skin cancer using cross-sectional questionnaire assessment, concluding that many non-dermatologists face challenges in recognizing and diagnosing different stages of skin cancer ​[3]​. Moreover, another study discussed the accuracy of non-dermatologists’ initial judgment on inpatient dermatologic conditions by comparing the non-dermatologist referring notes with the reviewing dermatologist response concluding that non-dermatologists misdiagnose many common and uncommon skin conditions respecting case rarity ​[4]​ and referral to a dermatologist is often warranted. A third study was conducted recently in 2020, where they used a retrospective methodology to analyze the data for patients who were referred or attended outpatient clinics seeking urgent care. This study showed that patients felt their condition needed immediate care but were unjustified​ [5]​. 

While there has been more research on the ability of non-dermatologist to diagnose skin cancer, few researchers covered the ability of non-dermatologists to recognize urgent skin diseases, which might be critical for general practitioners and other healthcare workers to recognize them early and provide the appropriate management.

Because very little data were conducted about this topic and the study was never done in our region, we aim to conduct an online assessment using a questionnaire assessing the ability of non-dermatologists to recognize urgent skin diseases at King Abdulaziz University Hospital (KAUH). As this data could be used to produce interventions that could optimize the quality of health care and help to prevent avoidable morbidities.

## Materials and methods

A prospective cross-sectional study was conducted at KAUH, a tertiary care center in Jeddah, Saudi Arabia. The institutional review board (IRB) has approved the study at KAUH (reference number: 171-22). Non-dermatologist physicians in KAUH were contacted through their verified emails, provided by the secretaries of each department and the academic affairs unit, between March and October 2022. Out of 300 physicians contacted, 161 agreed to participate in the study. An online questionnaire was conducted and reviewed by two dermatology consultants at KAUH and validated by the unit of biomedical ethics was sent to the participants.

The questionnaire was designed using Google Forms and consisted of two main sections, the first covering information about the physicians, including their: age, gender, specialty, and academic level [resident, specialist, consultant]. The second part assessed their ability to recognize different urgent dermatological conditions by showing them eight questions, each containing a brief case scenario about an urgent dermatological condition with a picture of the condition attached. Participants were required to answer the questions and assess on a scale from one to 10 how confident they were about their answers.

The study targeted non-dermatologist physicians and residents actively working at KAUH from March to October 2022; physicians working outside of KAUH and those inactive during the study period were excluded. SPSS (IBM Corp. Released 2010. IBM SPSS Statistics for Windows, Version 19.0. Armonk, NY: IBM Corp) was used for statistical analysis; continuous variables were described by calculating the mean and standard deviation, while numbers and percentages were used for categorical variables, chi-square test was used to evaluate the difference between the continuous and qualitative variables, respectively. A p-value of < 0.05 was considered significant.

## Results

One hundred sixty-one non-dermatology physicians completed the questionnaire; all the respondents were physicians from King Abdulaziz University Hospital and met the inclusion criteria. This study included 93 male physicians (57.8%) and 68 female physicians (42.2%). The mean age of the physicians who participated in the study was approximately 45 years. Out of all 161 responses to this questionnaire, there were 48 consultants (29.8%), 23 specialists (14.3%), and 90 residents (55.9%) that took part in the study.

Physicians of different specialties responded to the questionnaire. The specialties with the most responses were in the following order: Surgeons (25.2%), Medical Internists (21%), General Practitioners (17.3%), Pediatricians (12.4%), Obstetricians and Gynecologists (5.6%), Emergency Physicians (5%) and others (13%). Table 1 demonstrates the frequency and percentage of participants according to specialty.

**Table 1:** Frequency and percentage of participants according to specialty

specialty	frequency	percentage
Surgeons	41	25.2%
Internists	34	21%
General practitioners	28	17.3%
Pediatricians	20	12.4%
Obstetricians and Gynecologists	9	5.6%
Emergency physicians	8	5%
Others	21	13%

**Figure 1:**
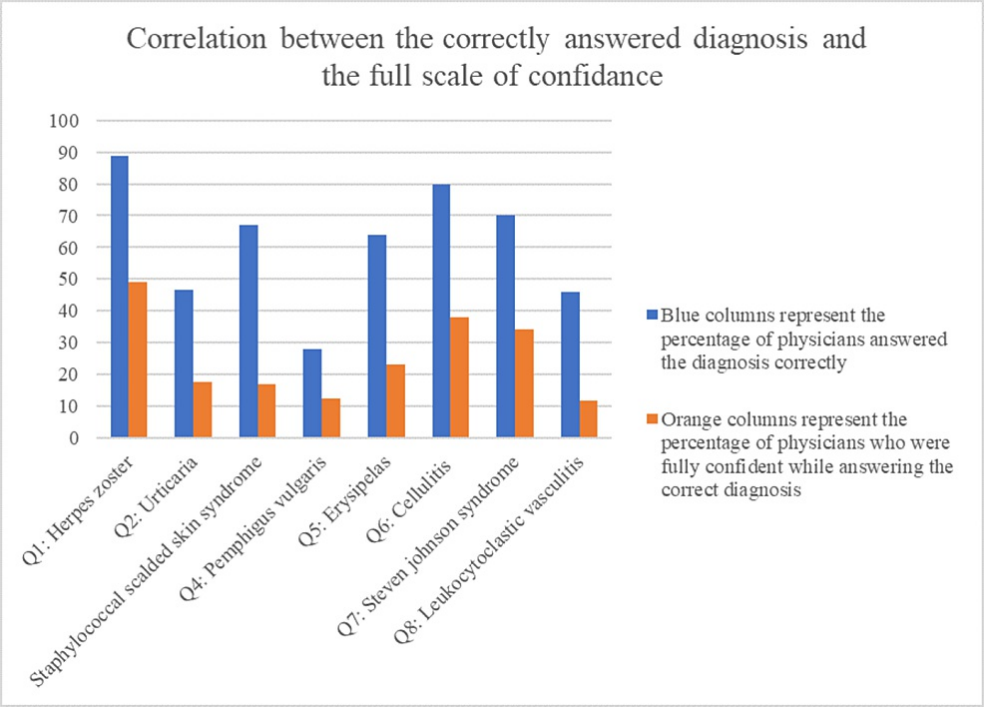
Correlation between the correctly answered diagnosis and the full scale of confidence

The most recognized urgent dermatological diseases and the least recognized ones were identified in the study and correlated with the confidence scale. Herpes zoster appeared to be the most recognizable urgent skin disease as it was correctly answered by 88.8% of the participants, Out of which 49.1% were fully confident about their answer. On the other hand, Pemphigus vulgaris was the least recognizable as it was only identified by 28% out of which 12.4% were fully confident. Furthermore, we found that the relation between the correct answer and the level of confidence about the diagnosis varied a lot in certain diseases, such as in staphylococcal scalded skin syndrome, where 67.1% of the participants answered it correctly when they were given the typical presentation of the condition, but only 16.8% of the physicians were sure and fully confident about their diagnosis. This shows that although some diseases were recognized correctly at a high percentage, only a few physicians were confident about their diagnosis. There was a considerable variation, which was inconsistent with the confidence level of the physicians when they answered it. as shown in Figure 1.

**Figure 2:**
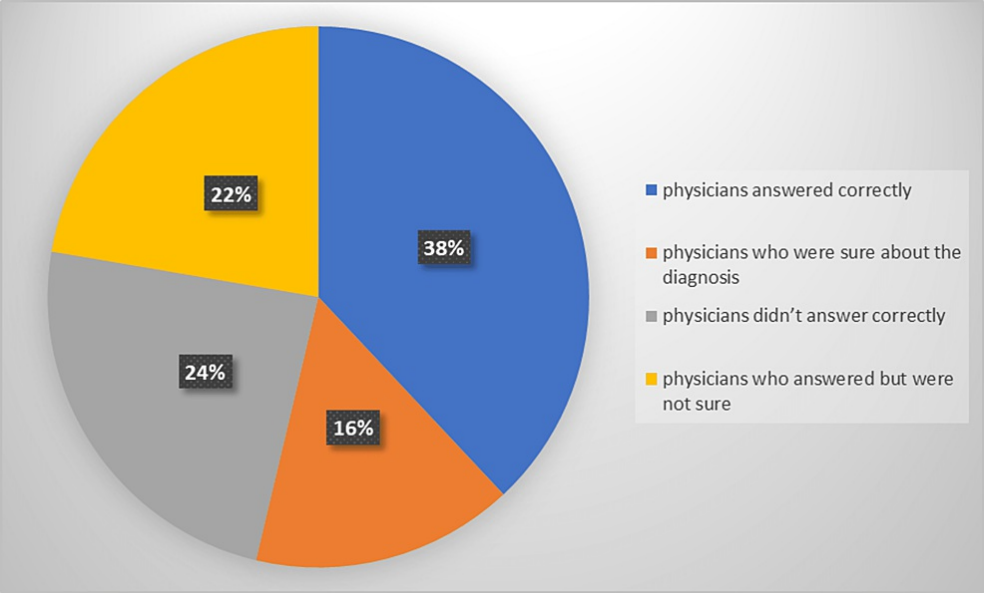
The percentage of making an accurate diagnosis for urgent skin diseases by non-dermatology physicians when given the typical presentation and scenario of the condition

Our study showed that the percentage of accuracy by non-dermatologists in diagnosing urgent skin diseases, given the typical presentation of the condition, was 61.33%. Nevertheless, the percentage decreased when it was recalculated with regard to the full confidence level to 25.3%. So a percentage of 36% expresses the probability of chance of having an accurate answer by rolling out and narrowing down the given choices. In addition, 38.67% of the physicians did not recognize those urgent skin conditions despite being shown the typical presentation of the disease. Figure 2 summarizes the accuracy percentages in diagnosing urgent dermatological conditions by non-dermatology physicians.

We found no statistical significance in recognizing urgent skin conditions between genders, P=0.417. Similarly, the educational level of the participants did not significantly affect the ability to answer correctly, P=0.281. Table 2 compares the accuracy of different specialties in diagnosing dermatological conditions.

**Table 2:** The accuracy rate in diagnosing different dermatological conditions by different specialties

Skin condition	Emergency Physicians	Family Physicians	Surgeons	Internists	Gynecologists	Pediatricians	Others
Herpes zoster	75	89.3	92.7	94.1	88.9	90	76.2
Urticaria	25	57	26.8	56	22.2	60	62
Staphylococcal Scalded Skin Syndrome	37.5	64.3	68.3	67.6	44.4	85	71.4
Pemphigus vulgaris	37.5	28.6	24.4	44.1	11	10	28.6
Erysipelas	87.5	75	68	70.6	55.6	45	42.9
Cellulitis	75	92.9	75.6	79.4	66.7	75	85.7
Steven Johnson syndrome	62.5	71.4	70.7	73.5	77.8	75	57
Leukocytoclastic vasculitis	50	39	44	56	33.3	65	28.6

It was revealed that 94.1% of internists were able to diagnose herpes zoster correctly, being the highest group. In contrast, 75% of emergency physicians could recognize it, the lowest group. Most pediatricians (60%) recognized urticaria being the highest group, while only 25% of emergency physicians and 22.2% of gynecologists diagnosed urticaria correctly, being the two lowest groups. Similarly, pediatricians were the best group to diagnose staphylococcal scalded skin syndrome (85%). While only 37.5% of emergency physicians diagnosed staphylococcal scalded skin syndrome correctly, being the lowest group. For Pemphigus vulgaris, 44% of internists correctly diagnosed it, 37.5% of emergency physicians recognized it, and only 10% of pediatricians could recognize it as the lowest group. For erysipelas, emergency physicians were the best group to recognize it (87.5%), while only 45% of pediatricians diagnosed erysipelas which was the lowest. For cellulitis, 92.9% of family physicians were able to diagnose it; being the group with the highest accuracy rate, 75% of emergency physicians recognized it. Gynecologists were the least to recognize cellulitis condition, with only 66.7%. For steven johnson syndrome, all specialty groups had similar accuracy in detecting it, with 77.8% of gynecologists and 62.5% of emergency physicians answering it correctly. Other specialties, including psychiatry and radiology, were the groups with the least accuracy in diagnosing steven johnson syndrome, as only 57.1% were able to recognize it. For leukocytoclastic vasculitis, 65% of pediatricians diagnosed it correctly, the group with the highest accuracy rate; 50% of emergency physicians recognized it, and 33% of gynecologists recognized that skin condition.

## Discussion

Dermatological disorders are some of the most prevalent medical conditions and comprise a large proportion of the global disease burden [6]. Early recognition and timely intervention are crucial to ensure the best outcome and prevent any complications [7], which is of particular significance in the case of urgent skin conditions. However, it is more common for patients to initially present to a primary care physician, internist or an emergency physician, who lack proper dermatology training [8,9]. This can lead to a delay in accurate diagnosis and proper treatment. Therefore, assessing the ability of non-dermatologist to recognize urgent skin diseases is crucial.

Our study finds that Herpes zoster virus is the most recognized urgent skin condition, with 88% of the participants answering the corresponding clinical case correctly with high confidence. This result is similar to a previous study conducted to assess the ability of family physicians to diagnose HZV, in which the clinical diagnosis made by the physicians was confirmed to be correct in more than 90% of the cases using serology as a reference [10]. A high index of knowledge about HZV is expected, as it is extensively taught to physicians during their medical school years. Furthermore, the Dermatological presentation is one part of a systemic illness involving other body organs [11]. This explains why doctors from different specialties have the comprehension necessary to recognize it.

Another well-identified condition is cellulitis which was answered by 80.1% of the participants correctly provided the correct answer. In contrast, a study performed to assess the diagnostic accuracy in patients admitted with cellulitis reported that around 33% of patients admitted and treated for cellulitis were misdiagnosed after a dermatologist reviewed their case [12]. The high level found in our study could be relayed to the fact that the question guided the doctors towards the diagnosis, with the study emphasizing that a dermatological condition is mostly the correct answer, which may not always be the case in an emergency or an outpatient setting, where other urgent differential diagnoses like deep venous thrombosis, abscesses, and septic joints [13] leading it being missed.

The least recognized condition in our study is Pemphigus vulgaris, as only 28% of doctors were able to diagnose it correctly; the low percentage of physicians being able to diagnose PV could be related to the low level of incidence, as reported by a study done in the southern region of Saudi Arabia in which the incidence of PV in the total population was reported at 0.16 cases/100,000 [14]. Another study by Hübner et al. [15], in which it had an incidence of 94.8 patients/million inhabitants amongst the German population. Thus, due to its rarity, and since it is almost an exclusive dermatological disorder, most doctors are not exposed to it during their school years and practice; thus, they are less likely to recognize it. A similar argument regarding the low level the medical and educational system grants dermatology could be made to explain the low level of recognition exhibited towards the remaining skin conditions, including Staphylococcal scalded skin syndrome, Urticaria, and Leukocytoclastic vasculitis. This is supported by the finding of a study done in California, to assess the ability of primary care residents showed that less than 40% of the participants believed their undergraduate curricula prepared to recognize and treat dermatological disorders [16].

The implementation of the confidence scale in our study further supports the theory that skin diseases are not well recognized by non-dermatologists, as our data shows that a randomly selected doctor from any specialty has only a 25% chance to diagnose an urgent skin disease with confidence. This is supported by a study assessing the diagnostic skills in dermatology reports that a substantial percentage of general practitioners do not include a provisional diagnosis in their referrals which may reflect a lack of confidence among them in diagnosing skin conditions [17].

The demographical data in our study, including the age and academic level, shows no statistical significance regarding the ability to diagnose urgent skin conditions, which reflects that dermatology is somewhat neglected even during the latter years of training programs, and doctors are not exposed to many cases prompting them to learn the proper ways of recognizing it even after they finish their training and are practicing consultants.

Study limitations include that the response to our questionnaire was limited in number, as not all doctors responded to the email. Not all dermatological emergencies have been included in our assessment. This study is done in a tertiary center. Hence, exposure to a lot of dermatological conditions aside from the outpatient setting is somewhat limited and does not reflect the level in centers which may have a more significant number of patients, we provided the participants with the typical book presentation of each case, which may not always occur, and thus the ability to diagnose might be lower than reported. The unavailability of a physical examination assessment is one of the limitations of our study.

## Conclusions

This study shows that there are some difficulties for physicians to recognize some urgent skin diseases which can affect the optimum health care for the patients. Moreover, more dermatology-focused courses are needed to strengthen the knowledge about dermatological diseases for current and future doctors during their undergraduate years. Urgent dermatological conditions should gain more attention during residency programs, especially for family and emergency physicians. Furthermore, future studies should be applied in multiple non-tertiary hospital centers aiming to include more significant numbers of Emergency physicians and general practitioners as they are more likely to face these conditions, incorporate atypical presentations, and assess the physicians' description of primary and secondary skin lesions in the medical report to prevent vague or misleading description in medical reporting, which may lead to unnecessary patient morbidity or neglect a necessary hospital admission.
